# Supplementary material for: Heparin-based hydrogel scaffolding alters the transcriptomic profile and increases the chemoresistance of MDA-MB-231 triple-negative breast cancer cells
Source: Biomater Sci. 2020 Feb 13;8(10):2786–96. doi: 10.1039/c9bm01481k (PMC7497406; doi:10.1039/c9bm01481k)
Supplement: Supplementary file 2 [file BM-008-C9BM01481K-s002.zip › Supplementary File 4/HP-BhydrogelvControl/Pathways/my_analysis.Gsea.1545201079907/HALLMARK_PI3K_AKT_MTOR_SIGNALING.html]

Details for gene set HALLMARK\_PI3K\_AKT\_MTOR\_SIGNALING[GSEA]

|  || Dataset | expr.class.cls#HYD\_versus\_CONTROL.class.cls#HYD\_versus\_CONTROL\_repos |
| Phenotype | class.cls#HYD\_versus\_CONTROL\_repos |
| Upregulated in class | CONTROL |
| GeneSet | HALLMARK\_PI3K\_AKT\_MTOR\_SIGNALING |
| Enrichment Score (ES) | -0.30687523 |
| Normalized Enrichment Score (NES) | -1.3183674 |
| Nominal p-value | 0.044444446 |
| FDR q-value | 0.07082992 |
| FWER p-Value | 0.45 |
Table: GSEA Results Summary

  

Fig 1: Enrichment plot: HALLMARK\_PI3K\_AKT\_MTOR\_SIGNALING      
 Profile of the Running ES Score & Positions of GeneSet Members on the Rank Ordered List

  

| PROBE | DESCRIPTION (from dataset) | GENE SYMBOL | GENE\_TITLE | RANK IN GENE LIST | RANK METRIC SCORE | RUNNING ES | CORE ENRICHMENT || 1 | RPTOR | na |  |  | 511 | 2.329 | 0.0069 | No |
| 2 | TRAF2 | na |  |  | 729 | 2.091 | 0.0258 | No |
| 3 | MKNK1 | na |  |  | 971 | 1.893 | 0.0405 | No |
| 4 | AKT1 | na |  |  | 1297 | 1.691 | 0.0479 | No |
| 5 | PLCG1 | na |  |  | 1500 | 1.575 | 0.0601 | No |
| 6 | TSC2 | na |  |  | 1754 | 1.468 | 0.0681 | No |
| 7 | NOD1 | na |  |  | 1791 | 1.446 | 0.0871 | No |
| 8 | PAK4 | na |  |  | 2019 | 1.353 | 0.0947 | No |
| 9 | GSK3B | na |  |  | 2118 | 1.307 | 0.1085 | No |
| 10 | DDIT3 | na |  |  | 2346 | 1.225 | 0.1143 | No |
| 11 | MAP2K3 | na |  |  | 2628 | 1.135 | 0.1160 | No |
| 12 | RAF1 | na |  |  | 3157 | 0.999 | 0.1028 | No |
| 13 | HRAS | na |  |  | 3805 | 0.850 | 0.0812 | No |
| 14 | FGF17 | na |  |  | 4130 | 0.784 | 0.0756 | No |
| 15 | STAT2 | na |  |  | 4243 | 0.764 | 0.0808 | No |
| 16 | E2F1 | na |  |  | 4422 | 0.730 | 0.0820 | No |
| 17 | MAPKAP1 | na |  |  | 4552 | 0.701 | 0.0854 | No |
| 18 | RPS6KA1 | na |  |  | 4592 | 0.693 | 0.0934 | No |
| 19 | CAB39 | na |  |  | 5013 | 0.623 | 0.0804 | No |
| 20 | PIN1 | na |  |  | 5032 | 0.621 | 0.0884 | No |
| 21 | EGFR | na |  |  | 5500 | 0.552 | 0.0719 | No |
| 22 | CDKN1B | na |  |  | 6043 | 0.481 | 0.0505 | No |
| 23 | ARHGDIA | na |  |  | 6491 | 0.417 | 0.0332 | No |
| 24 | SQSTM1 | na |  |  | 7012 | 0.359 | 0.0111 | No |
| 25 | AKT1S1 | na |  |  | 7444 | 0.313 | -0.0069 | No |
| 26 | ECSIT | na |  |  | 7833 | 0.268 | -0.0233 | No |
| 27 | NFKBIB | na |  |  | 7981 | 0.250 | -0.0274 | No |
| 28 | MKNK2 | na |  |  | 8109 | 0.246 | -0.0305 | No |
| 29 | CAMK4 | na |  |  | 9346 | 0.111 | -0.0936 | No |
| 30 | ACACA | na |  |  | 9410 | 0.104 | -0.0954 | No |
| 31 | TRIB3 | na |  |  | 9434 | 0.099 | -0.0951 | No |
| 32 | ITPR2 | na |  |  | 10019 | 0.038 | -0.1252 | No |
| 33 | MAPK10 | na |  |  | 10254 | 0.015 | -0.1372 | No |
| 34 | SMAD2 | na |  |  | 10612 | -0.018 | -0.1556 | No |
| 35 | AP2M1 | na |  |  | 10716 | -0.031 | -0.1606 | No |
| 36 | PDK1 | na |  |  | 10844 | -0.045 | -0.1666 | No |
| 37 | ATF1 | na |  |  | 11096 | -0.079 | -0.1785 | No |
| 38 | PPP2R1B | na |  |  | 11384 | -0.099 | -0.1921 | No |
| 39 | TNFRSF1A | na |  |  | 11403 | -0.101 | -0.1916 | No |
| 40 | MAPK8 | na |  |  | 11447 | -0.107 | -0.1923 | No |
| 41 | IRAK4 | na |  |  | 11729 | -0.142 | -0.2050 | No |
| 42 | CDKN1A | na |  |  | 11746 | -0.145 | -0.2037 | No |
| 43 | MYD88 | na |  |  | 12113 | -0.189 | -0.2201 | No |
| 44 | SFN | na |  |  | 12185 | -0.198 | -0.2210 | No |
| 45 | CALR | na |  |  | 12797 | -0.257 | -0.2492 | No |
| 46 | PITX2 | na |  |  | 12814 | -0.259 | -0.2463 | No |
| 47 | PIKFYVE | na |  |  | 12947 | -0.277 | -0.2492 | No |
| 48 | PIK3R3 | na |  |  | 13096 | -0.298 | -0.2527 | No |
| 49 | PTPN11 | na |  |  | 13303 | -0.326 | -0.2588 | No |
| 50 | PRKAR2A | na |  |  | 13336 | -0.327 | -0.2557 | No |
| 51 | CAB39L | na |  |  | 13466 | -0.348 | -0.2574 | No |
| 52 | PLA2G12A | na |  |  | 13728 | -0.392 | -0.2654 | No |
| 53 | CSNK2B | na |  |  | 14000 | -0.416 | -0.2736 | No |
| 54 | UBE2N | na |  |  | 14014 | -0.419 | -0.2682 | No |
| 55 | MAPK9 | na |  |  | 14479 | -0.487 | -0.2855 | No |
| 56 | RIPK1 | na |  |  | 14682 | -0.520 | -0.2885 | No |
| 57 | RALB | na |  |  | 14845 | -0.543 | -0.2892 | No |
| 58 | TIAM1 | na |  |  | 14991 | -0.561 | -0.2886 | No |
| 59 | PPP1CA | na |  |  | 15007 | -0.563 | -0.2813 | No |
| 60 | SLC2A1 | na |  |  | 15114 | -0.583 | -0.2784 | No |
| 61 | MAP3K7 | na |  |  | 15614 | -0.671 | -0.2948 | No |
| 62 | GRB2 | na |  |  | 15845 | -0.713 | -0.2966 | Yes |
| 63 | DUSP3 | na |  |  | 15932 | -0.730 | -0.2905 | Yes |
| 64 | RIT1 | na |  |  | 16098 | -0.769 | -0.2881 | Yes |
| 65 | ACTR2 | na |  |  | 16139 | -0.775 | -0.2790 | Yes |
| 66 | PFN1 | na |  |  | 16380 | -0.828 | -0.2796 | Yes |
| 67 | THEM4 | na |  |  | 16635 | -0.883 | -0.2801 | Yes |
| 68 | ARF1 | na |  |  | 16653 | -0.887 | -0.2682 | Yes |
| 69 | CFL1 | na |  |  | 16672 | -0.891 | -0.2562 | Yes |
| 70 | UBE2D3 | na |  |  | 16767 | -0.917 | -0.2479 | Yes |
| 71 | MAPK1 | na |  |  | 16876 | -0.948 | -0.2399 | Yes |
| 72 | CDK1 | na |  |  | 16949 | -0.970 | -0.2296 | Yes |
| 73 | CDK4 | na |  |  | 16963 | -0.974 | -0.2162 | Yes |
| 74 | PRKAA2 | na |  |  | 16975 | -0.978 | -0.2027 | Yes |
| 75 | PTEN | na |  |  | 17107 | -1.016 | -0.1949 | Yes |
| 76 | YWHAB | na |  |  | 17172 | -1.039 | -0.1832 | Yes |
| 77 | CDK2 | na |  |  | 17319 | -1.090 | -0.1751 | Yes |
| 78 | PRKAG1 | na |  |  | 17380 | -1.114 | -0.1621 | Yes |
| 79 | CLTC | na |  |  | 17555 | -1.181 | -0.1542 | Yes |
| 80 | EIF4E | na |  |  | 17756 | -1.264 | -0.1464 | Yes |
| 81 | HSP90B1 | na |  |  | 18044 | -1.410 | -0.1411 | Yes |
| 82 | NGF | na |  |  | 18094 | -1.437 | -0.1229 | Yes |
| 83 | TBK1 | na |  |  | 18386 | -1.630 | -0.1145 | Yes |
| 84 | RPS6KA3 | na |  |  | 18422 | -1.654 | -0.0925 | Yes |
| 85 | ACTR3 | na |  |  | 18460 | -1.682 | -0.0701 | Yes |
| 86 | NCK1 | na |  |  | 18624 | -1.812 | -0.0525 | Yes |
| 87 | ARPC3 | na |  |  | 18626 | -1.815 | -0.0263 | Yes |
| 88 | RAC1 | na |  |  | 18642 | -1.836 | -0.0006 | Yes |
| 89 | CXCR4 | na |  |  | 18827 | -2.050 | 0.0194 | Yes |
Table: GSEA details [plain text format]

  

Fig 2: HALLMARK\_PI3K\_AKT\_MTOR\_SIGNALING      
 Blue-Pink O' Gram in the Space of the Analyzed GeneSet

  

Fig 3: HALLMARK\_PI3K\_AKT\_MTOR\_SIGNALING: Random ES distribution      
 Gene set null distribution of ES for **HALLMARK\_PI3K\_AKT\_MTOR\_SIGNALING**
